# Single ion fluorescence excited with a single mode of an UV frequency comb

**DOI:** 10.1038/s41467-017-00067-9

**Published:** 2017-06-29

**Authors:** Akira Ozawa, Josue Davila-Rodriguez, James R. Bounds, Hans A. Schuessler, Theodor W. Hänsch, Thomas Udem

**Affiliations:** 1 Max-Planck-Institut für Quantenoptik, Hans-Kopfermann-Strasse 1, 85748 Garching, Germany; 2 Department of Physics and Astronomy, Texas A&M University, College Station, Texas 77843 USA; 3 National Institute of Standards and Technology (NIST), 325 Broadway, Boulder, 80305 CO USA

## Abstract

Optical frequency combs have revolutionized the measurement of optical frequencies and improved the precision of spectroscopic experiments. Besides their importance as a frequency-measuring ruler, the frequency combs themselves can excite target transitions (direct frequency comb spectroscopy). The direct frequency comb spectroscopy may extend the optical frequency metrology into spectral regions unreachable by continuous wave lasers. In high precision spectroscopy, atoms/ions/molecules trapped in place have been often used as a target to minimize systematic effects. Here, we demonstrate direct frequency comb spectroscopy of single ^25^Mg ions confined in a Paul trap, at deep-UV wavelengths. Only one mode out of about 20,000 can be resonant at a time. Even then we can detect the induced fluorescence with a spatially resolving single photon camera, allowing us to determine the absolute transition frequency. The demonstration shows that the direct frequency comb spectroscopy is an important tool for frequency metrology for shorter wavelengths where continuous wave lasers are unavailable.

## Abstract (web summary)

Frequency combs are useful tools in high precision measurement including atomic transitions and atomic clocks. Here the authors demonstrate direct frequency comb spectroscopy to shorter wavelengths by probing a transition frequency in a trapped Mg+ ion using a single mode of a UV frequency comb.

## Introduction

For almost 20 years, optical frequency combs have enabled significant progress in high precision optical frequency metrology. A prominent example is the frequency of the 1*S*-2*S* two-photon transition in atomic hydrogen, which has been measured with a fractional uncertainty of 4.1 × 10^−15^ ^1, 2^. Another milestone are the atomic clocks that have been improved by orders of magnitude by using optical transitions^3^. Many other fields such as astronomy^4^, low-noise frequency synthesis^5^, and trace gas analysis^6^ have benefited enormously from frequency combs. Frequency combs have also allowed control of the carrier-envelope phase of short pulses^7^ and by that enabled experiments with single attosecond pulses^8^. Thanks to the short pulse nature of the generating pulse train non-linear interactions are efficient so that frequency combs can be converted to much shorter wavelengths where continuous wave (cw) lasers are not available. While commercially available crystals are limited by transparency and phase matching requirements to about *λ* > 190 nm and not yet commercially available KBBF crystals improve this limit to approximately *λ* > 150 nm^9, 10^, high harmonic generation (HHG) can reach well into the X-ray regime^11^. Even though there is very low power with very low repetition rate generated at these most extreme wavelengths, frequency combs with a discernable mode spacing and μW power level can now be produced at least in the extreme ultra violet (EUV)^12^. The HHG process allows for a coherence time of 1 s provided that a suitable driving laser is used^13^. This compares well with the state-of-the-art cw lasers in the visible.

A coherent pulse train from a mode locked laser corresponds to a frequency comb in the spectral domain. The frequencies of the modes of the comb, either at the fundamental or upconverted wavelength, are given by *f*_*n*_ = *nf*_r_ + *f*_o_ with the repetition frequency (=mode spacing) *f*_r_, which lies in the radio frequency domain. Knowing *f*_r_ and the radio frequency offset *f*_o_, the optical frequencies of the modes *f*_*n*_ may be computed after the large integer *n* has been determined^14^. While this property has been used for optical frequency metrology, it is possible to employ the modes for direct frequency comb spectroscopy (DFCS). Using a single mode of a frequency comb on the dipole allowed Cs D lines, V. Gerginov and co-workers could perform DFCS on an atomic beam^15^. While this is a beautiful demonstration, it also revealed some problems: large ac Stark shifts and high levels of stray light are expected due to the large number of spectator modes. These disadvantages are absent when driving a pure two-photon transition with a frequency comb, i.e. without near-resonant intermediate levels. In that case the modes contribute pairwise to the excitation, which leads to a transition rate that is given by the total power of all modes, while retaining the line width of an individual mode^16, 17^. In fact the first application of DFCS in 1978 was of this kind^18^. The original idea of Ye.V. Baklanov and V.P. Chebotayev for two-photon DFCS was to enter a new region of shorter wavelength not accessible with cw lasers^19^. However, in this case perfect cancellations of the first order Doppler effect is more difficult than with cw lasers. Due to their large band width, counter propagating photons may not have exactly opposite Doppler shifts leading to a type of time of flight broadening^20, 21^. Other problems emerge when a diverging atomic beam is used in conjunction with chirped pulses^22^. Trapping and holding the atoms or ions in place for interrogation is the solution to these issues that all modern optical clocks employ.

T.M. Fortier and coworkers were the first to demonstrate DFCS on cold calcium atoms in a magneto optical trap using the narrow intercombination line at 657 nm^23^. Later A.L. Wolf et al. were exciting clouds of calcium ions in a trap^24^ with a frequency comb. Recently we could show how to keep a trapped ion refrigerated by the cooling action of an individual mode of an UV comb^25^. However, in the previous work^25^, the systematic effects were not studied and the suitability of the method for precision spectroscopy was not ascertained.

Here we utilize a single mode from an ultraviolet frequency comb at 280 nm to excite the 3*s*_1/2_−3*p*_3/2_ transition in a single trapped ^25^Mg ion. Individual trapped Mg^+^ ions are used because large clouds of ions are subject to strong systematic shifts. The excitation is detected by observing the induced fluorescence with a spatially resolving single photon camera. Although the expected amount of fluorescence is extremely small since only one mode of the comb can contribute to a signal, we determine the absolute frequency of this transition with improved uncertainty over previous measurements using a cw laser. The technique can be extended straightforwardly to other transitions in different ions. It holds particular interest for future experiments with high harmonic frequency combs in the EUV regions, where no cw lasers are available. In this way, a vast spectral territory will become accessible to precision laser spectroscopy for the first time. This possibility would be for the benefit of fundamental physics as all hydrogen-like ions, like He^+^ have their metrologically relevant transitions^26^ there and beyond. DFCS in the EUV has only been performed with fast atoms on strong broadband transitions^27–29^ that have a short excited lifetime and are of limited metrological interest. In our demonstration, it is shown that high precision DFCS using single trapped ions is indeed possible without introducing significant distortions at deep-UV wavelengths. This is a prerequisite for future precision measurements including EUV shelving^30^.

## Results

### Ion trapping and UV frequency comb generation

**Fig. 1:**
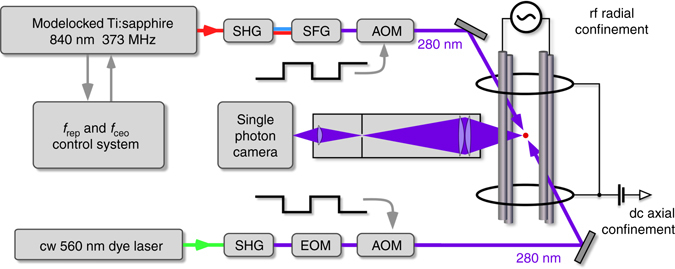
Experimental setup schematic. A frequency doubled cw dye laser is used for laser cooling trapped Mg ions on the 3*s*_1/2_−3*p*_3/2_ transition. The same transition is interrogated with an individual mode of the UV frequency comb that derives from the third harmonic of a Ti:sapphire mode locked laser and is spatially overlapped with the cw laser. The two lasers are alternatingly turned on for 2 ms with two acousto optic modulators (AOM). Thanks to its lower power, detuning dependent residual heating/cooling due to the near resonant mode of the comb is largely suppressed (see text). The fluorescence signal is detected by spatially resolved single photon camera during the bright times of the comb i.e. the dark periods of the cooling laser. The ion trap consists of four rf-electrodes, two pairs of ring electrodes for axial confinement and four rod-like dc-electrodes (not shown) for stray field cancellation. *SHG* second-harmonic generation stage, *SFG* sum frequency generation stage, *EOM* electro-optical-modulator

**Fig. 2:**
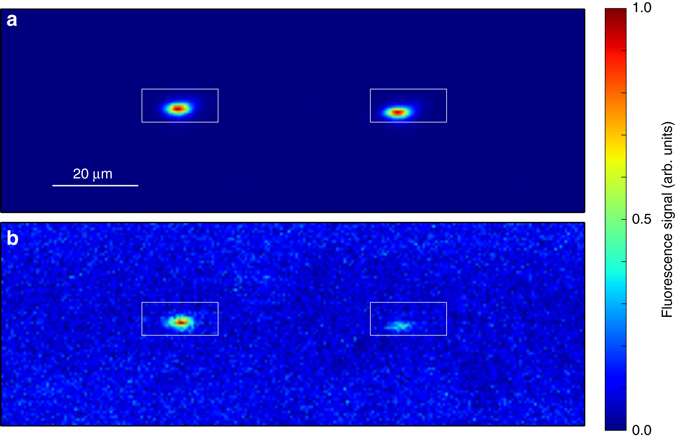
Ion images. Typical ion images for **a** cooling and **b** spectroscopy periods. We use one or two ions for spectroscopy. When two ions are trapped, the illumination across them is not exactly uniform because their extension exceeds the tightly focused laser beams waists of the frequency comb and cooling laser. The fluorescence signal is recorded within the ROI denoted by the white rectangles. This area is set to accommodate the average ion position, so it does not necessarily center the ions on each run. Note that the images are normalized independently for **a** and **b**

Our experimental apparatus is schematically shown in Fig. 1. Here we briefly describe the setup, while further details can be found in our previous publications^25, 26^. Ions are trapped in a linear radiofrequency (rf) quadrupole trap driven at 22 MHz, with radial and axial secular frequencies of *ω*_r_≈2*π* × 1 MHz and *ω*_ax_≈2*π* × 45 kHz, respectively. To minimize the micromotion and compensate for stray fields in the trapping region, suitably chosen voltages are applied to additional rods surrounding the rf-electrodes. The ion loading process starts with neutral Mg vapor from an atomic oven which is resonantly photo-ionized using a pulsed and frequency doubled dye laser at 285 nm that is pumped with the second harmonic of a Q-switched Nd:YAG laser. Typically we load one or two ions by cooling on the 280 nm 3*s*_1/2_−3*p*_3/2_ transition. The cooling radiation is obtained by frequency doubling a single-frequency dye (Rhodamine-19) laser at 560 nm. When an isotopically pure ion chain is desired, the loading is repeated until a pure sample of, for example, ^25^Mg^+^ is loaded. The probability of loading a particular isotope can be increased by tuning the photo-ionization laser to the optimum frequency. The frequency comb is obtained from a ~840 nm Ti:sapphire mode-locked ring laser that operates at a repetition rate of 373 MHz with a spectral bandwidth (FWHM) of ~20 nm and an average output power ~400 mW. The repetition rate is detected with a high-speed photodiode and stabilized via feedback to an intra-cavity piezoelectric transducer-mounted mirror. Approximately 40% of the output power is sent into an *f*−2*f* interferometer to detect the offset frequency *f*_o_^14^. The offset frequency is stabilized to a radio frequency reference by feeding back to the rf-drive of an acousto-optic modulator (AOM) that drains power from the pump beam of the Ti:sapphire mode-locked laser. Both comb parameters (*f*_r_ and *f*_o_) are locked to oscillators which are phase-locked to a GPS-calibrated hydrogen-maser, ensuring traceability of the frequency of each comb-line. The remaining ~240 mW of the comb power at 840 nm from the oscillator is first focused into a 1-mm thick beta barium borate (BBO) crystal to generate the second harmonic at 420 nm. The unconverted fundamental light is temporally overlapped with the 420 nm pulse train and focused in a second BBO crystal to generate the sum-frequency at 280 nm (see also^25^). The 280 nm pulse-train is sent through an AOM and focused into the trap. We typically deliver 40–80 μW at the ions. We estimate the power-per-comb-line at the few nW level, which provides an intensity on the order of 0.5 mW cm^−2^, which corresponds to 0.2% of the saturation intensity. The fluorescence from the ions is collected by a four-lens condenser (B. Halle Nachfl., N.A. ≈ 0.25). An iris limits the aperture of the condenser to obtain nearly diffraction limited images. A ×13 microscope objective images the intermediate focus onto a single photon camera (Quantar Technology, Mepsicron II) with a total magnification of about 100. To suppress the background light level a solar blind color filter is placed in front of the detector. In a conventional spectroscopy experiment with a cw laser and ions in the weak-binding regime, detuning-dependent heating and cooling gives rise to strong lineshape asymmetries that rule out any precision experiment. The same is true for DFCS of a single photon transition as shown in our previous work^25^. In order to keep the ions cold during the spectroscopy, sympathetic cooling can be employed. Such sympathetic cooling will be necessary for the envisioned EUV experiments^26, 31, 32^. Sympathetic cooling requires that the cooling and spectroscopy beams be focused on different ions and is thus less appropriate for Coulomb crystals composed of a very small number of ions. Since a cw cooling laser is available in the present experiments, we rapidly switch cooling and spectroscopy laser as demonstrated before^33^. The fixed frequency cooling laser and the frequency comb are alternatingly applied to the same ions. AOMs are used for switching the beams with a rise/fall time of <1 μs. The cooling and spectroscopy periods are both 2 ms. The lasers are both focused to *w*_0_ ~ 20 μm. Due to the small angle between the lasers and the trap axis all ions in the chain are illuminated to some extent. For the center ion we estimate the Rabi frequency to be 2*π* *×* 29 MHz for the cooling laser and 2*π* *×* 1.4 MHz for one mode of the spectroscopy laser. The arrival times of the fluorescence counts are time-tagged and only those events registered within the spectroscopy periods are evaluated as the spectroscopy signal. The pulse sequence and data acquisition are controlled by a real-time I/O system (ADwin Pro-II). Examples of fluorescence images of the ion chain recorded during cooling and spectroscopy periods are shown in Fig. 2a, b respectively.

### DFCS by fluorescence detection

**Fig. 3:**
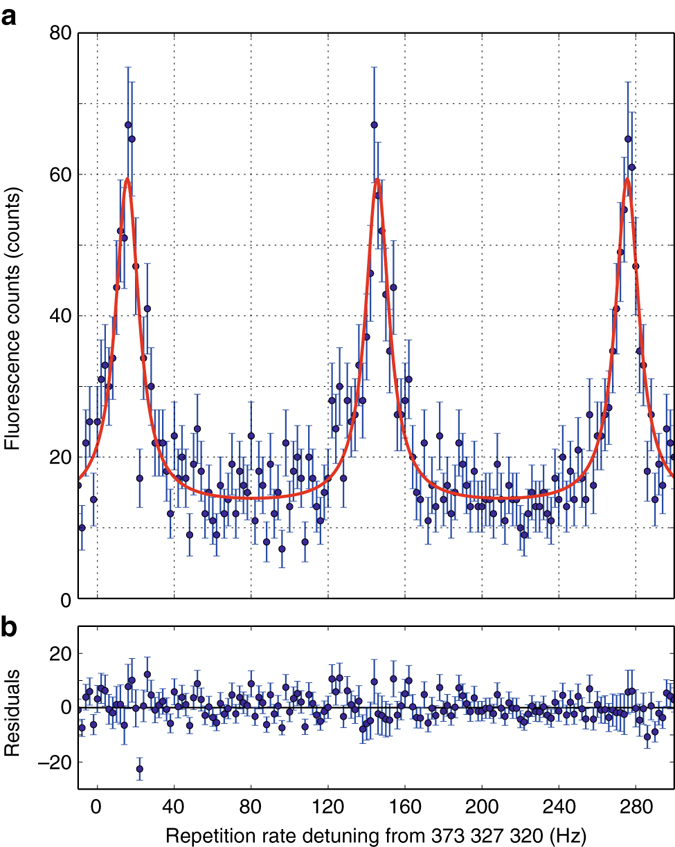
Typical spectroscopy signal. **a** The longitudinal modes of the frequency comb are scanned over the ^25^Mg^+^ D_2_ resonance by changing the repetition rate while the fluorescence from an individual ion is collected. The signal is periodic just as the comb is. To convert the detuning of the repetition rate to optical frequency one needs to multiply with the mode number which is *n* = 2,871,702 for the central peak. The photon count rate at the peak of resonance was ~7 Hz. The signal was accumulated to give a larger signal count. The data are fitted with a series of Lorentzians as indicated by a *solid red curve* and the fit residuals are shown in **b**. The linewidth of the signal peaks is about 43 MHz in good agreement with the natural linewidth of 41.8 MHz^40^. The error bars are taken as the square root of the signal counts assuming Poissonian statistics for both **a** and **b**

The comb modes are scanned over the resonance by changing the repetition rate of the frequency comb while recording frames with images of the ions. For data analysis a region of interest (ROI) is defined around each ion and total fluorescence counts are extracted as a function of the absolute frequency. The latter is determined with the help of the frequency comb which is referenced to the hydrogen maser. The mode number is determined to be consistent with previous measurements performed with a cw laser^26, 31^. A typical spectroscopy signal is shown in Fig. 3. Assuming Poissonian statistics, the error bars in Fig. 3 are taken as the square root of the signal counts.

## Discussion

**Fig. 4:**
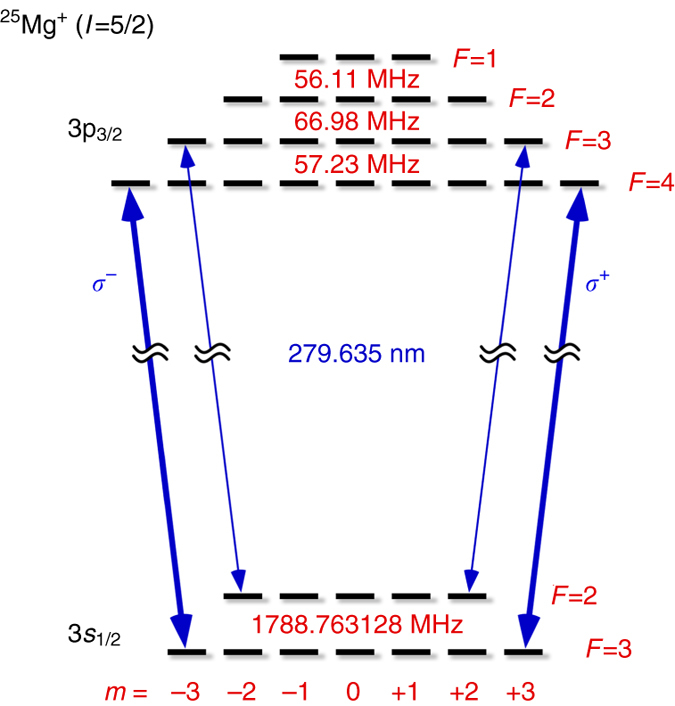
The level scheme of ^25^Mg^+^. The isotope ^25^Mg^+^ used in this demonstration has a nuclear spin of *I* = 5/2. The resulting hyperfine structure denoted by (*F*,*m*) is calculated using the usual *A* and *B* factors taken from^38^ for the ground state (*A* = −596.254376(54) MHz) and from^39^ for the excited state (*A* = −18.89 MHz, *B* = 22.91 MHz). The lowest lying hyperfine level is 745.317970(68) MHz and 65.11 MHz below the hyperfine centroid for the 3*s*_1/2_ and the 3*p*_3/2_ states, respectively. The cycling transitions (*thick arrows*) are driven with either *σ* ^+^ or *σ* ^−^ polarization. The *F* = 2 ground state, that is populated by off resonant excitation, is kept unpopulated by repumper sidebands modulated on the laser (*thin arrows*)

The repeating peaks of the signal shown in Fig. 3 reflect the comb-mode structure of the excitation laser. The signal contrast defined as a ratio between fluorescence counts at the peak and the base-line is ~3.6. This contrast deteriorates to ~0.01 without a properly defined ROI., i.e., with the full frame fluorescence signal. Exciting the transition with a multiple of comb lines, on and off resonant, does produce beatings in the fluorescence signal at *f*_r_ and harmonics. Since we detect the signal for several seconds, any coherent line distortion due to that beating is averaged out. Therefore the data are fitted with a series of Lorentzians, where we assume that the noise of the fluorescence counts is given by shot noise only. Quantum interference with the off-resonant D_1_-line can be neglected as the line separation measured in units of the line width is large enough^34^. However, the spectrum of our frequency comb is broad enough to also address the D_1_-line. In that case it folds in between the repeated D_2_-lines shown in Fig. 3 and distorts the simple periodic Lorentzian shape. We avoid this by employing the cycling transitions with either *σ* ^+^ or *σ* ^−^ polarization as sketched in Fig. 4. There is no cycling transition connecting the ground state and the 3*p*_1/2_ state.

**Fig. 5:**
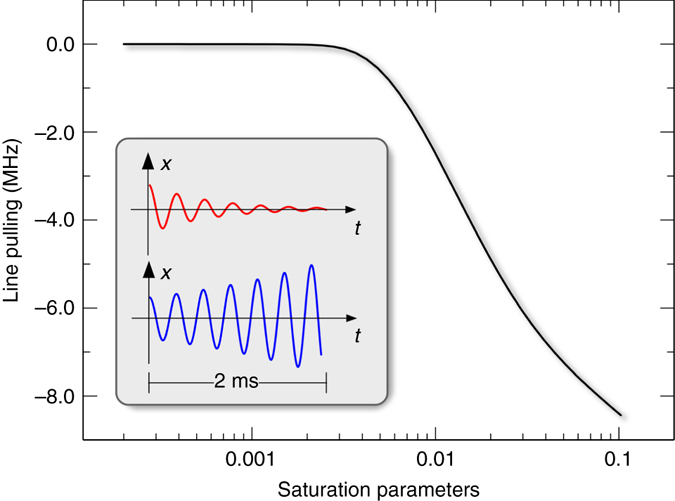
Line pulling due to heating/cooling of the spectroscopy laser. The radiation pressure of the cooling laser misplaces the ion out of its potential minimum by *x* ≈ 2 μm. Sudden termination of the cooling laser sets the ion in motion. Depending on the detuning of the spectroscopy laser this oscillation is either damped or amplified (see *inset*, not to scale). The dynamics is modeled numerically and the fluorescence signal is determined for different detunings of the spectroscopy laser. The resulting slightly asymmetric line shape is then fitted by a Lorentzian to measure the line pulling due to this effect. The saturation parameter measures the laser intensity *I* through *s* = *I*/*I*_s_ with *I*_s_ = 250 mW cm^−2^

The absolute frequency measurement is subject to several other systematic effects. Without the interleaving cooling laser, fluorescence would be seen only for red detuning because the large orbits of the heated ions essentially eliminates the fluorescence for blue detuning. Even with the cooling laser the ion motion is either amplified or damped during the spectroscopy periods causing a small asymmetry of the line shape. To estimate this effect we numerically simulate the detuning dependent ion trajectories and compute the florescence signal. We then determine the line center by fitting a Lorentzian to these data. This operational quantification of the line shift is adapted to a real data analysis. The result as a function of spectroscopy laser intensity is shown Fig. 5. It is interesting to note that this systematic error has a threshold behavior. With the above given laser parameters we estimate the saturation parameter to be less than 0.002 so that the line shift is less than 14 kHz. To compensate the Zeeman shift, we use Helmholtz coils to first cancel the residual magnetic fields at the trapping region. A small magnetic field is then applied along the spectroscopy laser beam to define a quantization axis. Microwave spectroscopy on the 3*s*_1/2_(*F* = 3) → 3*s*_1/2_(*F* = 2) transition reveals the applied magnetic field strength to be ~ 1.8 Gauss. The polarization of the cooling laser is set to be *σ* ^+^ using the 3*s*_1/2_(*F*, *m*) = (3,3) → 3*p*_3/2_(*F*,*m*) = (4,4) cycling transition (see Fig. 4). Frequency modulation sidebands are imposed on the cooling laser with an electro–optic modulator to prevent population trapping into the *F* = 2 ground state. Hence, during the cooling periods, the ions are prepared into 3*s*_1/2_(*F*, *m*) = (3, 3) ground state^35, 36^. During the spectroscopy period, the 3*s*_1/2_(*F*, *m*) = (3,3) → 3*p*_3/2_(*F*,*m*) = (4,4) transition is then probed with the *σ* ^+^ polarized frequency comb. The Zeeman shift is equal in magnitude but opposite in sign for the (*F*,*m*) = (3,−3) → (*F*,*m*) = (4,−4) component driven (and cooled) with *σ* ^−^ polarization. We cancel the residual Zeeman shift by averaging the results for the two polarizations. A total of 48 line scans leaves us with a statistical uncertainty of 0.45 MHz. Imperfect circular polarization contributes 0.3 MHz to the uncertainty, determined by performing measurements with intentionally misaligned polarization. The ac-Stark shift of the ground and 3*p*_3/2_ states induced by off-resonant comb-modes is estimated to be less than 7 kHz. The comb spectrum also covers the 3*p*_3/2_ → 3*d*_5/2_ transition and therefore gives rise to an ac-Stark shift of the 3*p*_3/2_ state. In addition, the fluorescence from the 3*d*_5/2_ states may distort the line-shape of the 3*s*_1/2_ → 3*p*_3/2_ spectroscopy signal. We used the appropriate three-level optical Bloch equations to model these effects and found them to contribute with less than 0.1 MHz. Considering all of these effects, the total uncertainty is estimated to 0.7 MHz. Other systematic shifts like the dc-Stark shift from the trapping fields and 2nd order Doppler shift are negligible. We obtain 1072085233.5(0.7) MHz for the 3*s*_1/2_(*F* = 3)→3*p*_3/2_(*F* = 4) transition. This is the smallest reported uncertainty on this transition, showcasing the ability of the frequency comb to perform precision spectroscopy^31, 37^. Using the hyperfine constants from the literature^38, 39^, the centroid frequency of the ^25^Mg^+^ D_2_ line is found to be 1072084553.3(0.7) MHz, where we assume that the hyperfine constants^39^ are as accurate as the number of digits shown. It should be noted that Table 2^39^ gives the magnitude of A and B factors only such that the sign of the A factors has to be flipped (C. Sur, personal communication). This value is in good agreement with a recent measurement that uses the new method of quantum decoherence spectroscopy^37^. The uncertainty of the latter measurement is not intrinsic in the method, but was limited by the wavelength measurement. Our value also compares well with a previous determination from the ^24^Mg^+^ D_2_ transition frequency using a theoretical value for the isotope shift^31^. Table 1 summarizes our result in comparison with previous determinations of this transition.

**Table 1:** Comparison of the ^25^Mg^+^ D_2_ line transition frequencies

Reference	^25^Mg^+^ D_2_ line in MHz
This work	1 072 084 553.3 (0.7)
Batteiger et al.^31^	1 072 084 555 (19)
Clos et al.^37^	1 072 084 547 (5)

The transition frequency measured in this work is shown in comparison to previous determinations along with their uncertainty

In summary, DFCS using an individual mode and individual ions is demonstrated with the 3*s*_1/2_−3*p*_3/2_ transition of ^25^Mg^+^. Spatially resolved detection suppresses the contribution of the scattering background and enhances the contrast of the fluorescence signal by more than two orders of magnitude. The obtained transition frequency is confirmed to be consistent with conventional measurements using cw lasers. The method is simple and versatile and can be applied to a wide range of transitions and significantly shorter wavelengths. It does not require a special level structure that would be required for other sensitive detection methods such as shelving and quantum decoherence detection.

### Data availability

The data that support the findings of this study are available from the corresponding author upon reasonable request.
